# Malat1 deficiency prevents neonatal heart regeneration by inducing cardiomyocyte binucleation

**DOI:** 10.1172/jci.insight.162124

**Published:** 2023-03-08

**Authors:** Galip S. Aslan, Nicolas Jaé, Yosif Manavski, Youssef Fouani, Mariana Shumliakivska, Lisa Kettenhausen, Luisa Kirchhof, Stefan Günther, Ariane Fischer, Guillermo Luxán, Stefanie Dimmeler

**Affiliations:** 1 Institute of Cardiovascular Regeneration, Center of Molecular Medicine, and; 2 Faculty of Biological Sciences, Goethe University, Frankfurt, Germany.; 3 German Center for Cardiovascular Research DZHK, Berlin, Germany, partner site Frankfurt Rhine-Main, Germany.; 4 Cardiopulmonary Institute, Goethe University, Frankfurt, Germany.; 5 Max Planck Institute for Heart and Lung Research, Bioinformatics and Deep Sequencing Platform, Bad Nauheim, Germany.

**Keywords:** Cardiology, Cell Biology, Cardiovascular disease, Noncoding RNAs

## Abstract

The adult mammalian heart has limited regenerative capacity, while the neonatal heart fully regenerates during the first week of life. Postnatal regeneration is mainly driven by proliferation of preexisting cardiomyocytes and supported by proregenerative macrophages and angiogenesis. Although the process of regeneration has been well studied in the neonatal mouse, the molecular mechanisms that define the switch between regenerative and nonregenerative cardiomyocytes are not well understood. Here, using in vivo and in vitro approaches, we identified the lncRNA Malat1 as a key player in postnatal cardiac regeneration. Malat1 deletion prevented heart regeneration in mice after myocardial infarction on postnatal day 3 associated with a decline in cardiomyocyte proliferation and reparative angiogenesis. Interestingly, Malat1 deficiency increased cardiomyocyte binucleation even in the absence of cardiac injury. Cardiomyocyte-specific deletion of Malat1 was sufficient to block regeneration, supporting a critical role of Malat1 in regulating cardiomyocyte proliferation and binucleation, a landmark of mature nonregenerative cardiomyocytes. In vitro, Malat1 deficiency induced binucleation and the expression of a maturation gene program. Finally, the loss of hnRNP U, an interaction partner of Malat1, induced similar features in vitro, suggesting that Malat1 regulates cardiomyocyte proliferation and binucleation by hnRNP U to control the regenerative window in the heart.

## Graphical abstract

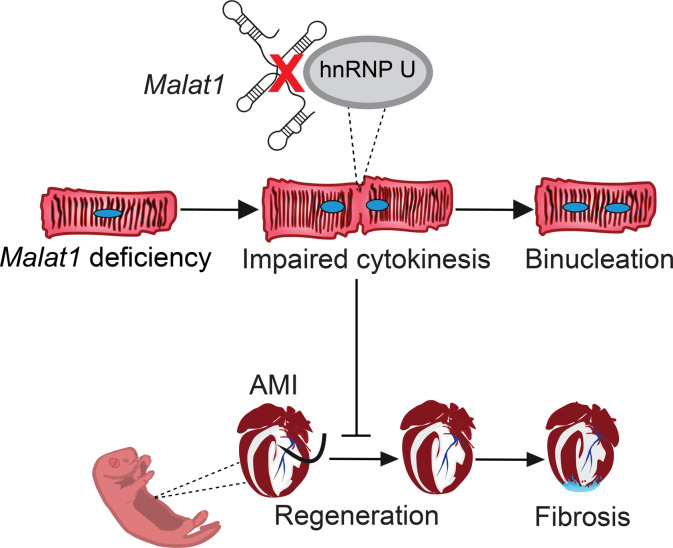

## Introduction

During the first week of life, mice are able to regenerate injured myocardium (1, 2). Similar to other species with regeneration capabilities, regeneration of the murine postnatal heart is achieved by the proliferation of existing cardiomyocytes (1, 3, 4). The infiltration of proregenerative immune cells (5), angiogenesis and arteriogenesis (6), and innervation of the cardiac tissue (7) contribute to this transient regenerative capacity. After this period, even though there is DNA synthesis in cardiomyocytes, it is mostly associated with multinucleation (8). Particularly, the emergence of polyploid or binucleated cardiomyocytes has been proposed as the cause for the loss of the regenerative capacity of zebrafish and murine postnatal regeneration (9, 10). Also, in larger mammals and humans, cardiomyocytes are transitioning from a mononucleated and proliferative state to multinucleation throughout life (11–13). Several studies have addressed the mechanisms underlying the decline in regeneration and have reported transcriptional and metabolic mechanisms accounting for the loss of cardiomyocyte proliferation (14). The metabolic reprogramming of cardiomyocytes by ErbB2 is essential for cardiomyocyte proliferation in the regenerative heart (15, 16). Furthermore, an active form of the Hippo pathway effector Yap promotes cardiac regeneration by activating the expression of embryonic and proliferative gene expression programs (17). Also, small noncoding microRNAs such as miR-15 (2), miR-199 (18), and miR-34a (19) regulate cardiomyocyte proliferation.

The human genome contains from 16,000 up to 100,000 long noncoding RNAs (lncRNAs) (20, 21). lncRNAs are defined as transcripts longer than 200 nucleotides that are not translated into proteins (22). They can regulate the expression of other genes (23) and are expressed in a cell type–specific manner (22). In cardiomyocytes, different lncRNAs like CRRL, CAREL, CPR, or LncDACH1 can regulate proliferation, while others like CARL, Sirt1, Meg3, or H19 have been reported to regulate cardiomyocyte apoptosis, affecting cardiac injury and repair (reviewed in ref. 24). Malat1 is a lncRNA that is highly expressed in different organs, including the heart (25, 26). Previous studies have shown that Malat1 regulates the balance between proliferation and migration of endothelial cells, controlling vessel growth (27), and that its deficiency induces atherosclerotic lesion formation in mice (28). In the adult mouse, Malat1 has been shown to induce cardiomyocyte apoptosis (29) and fibrosis (30) after myocardial infarction (MI). Furthermore, Malat1 has been discussed as a potential biomarker after acute MI (31, 32). However, its role in neonatal heart regeneration has not been studied to the best of our knowledge.

## Results

### Malat1 deficiency prevents postnatal heart regeneration.

**Figure 1:**
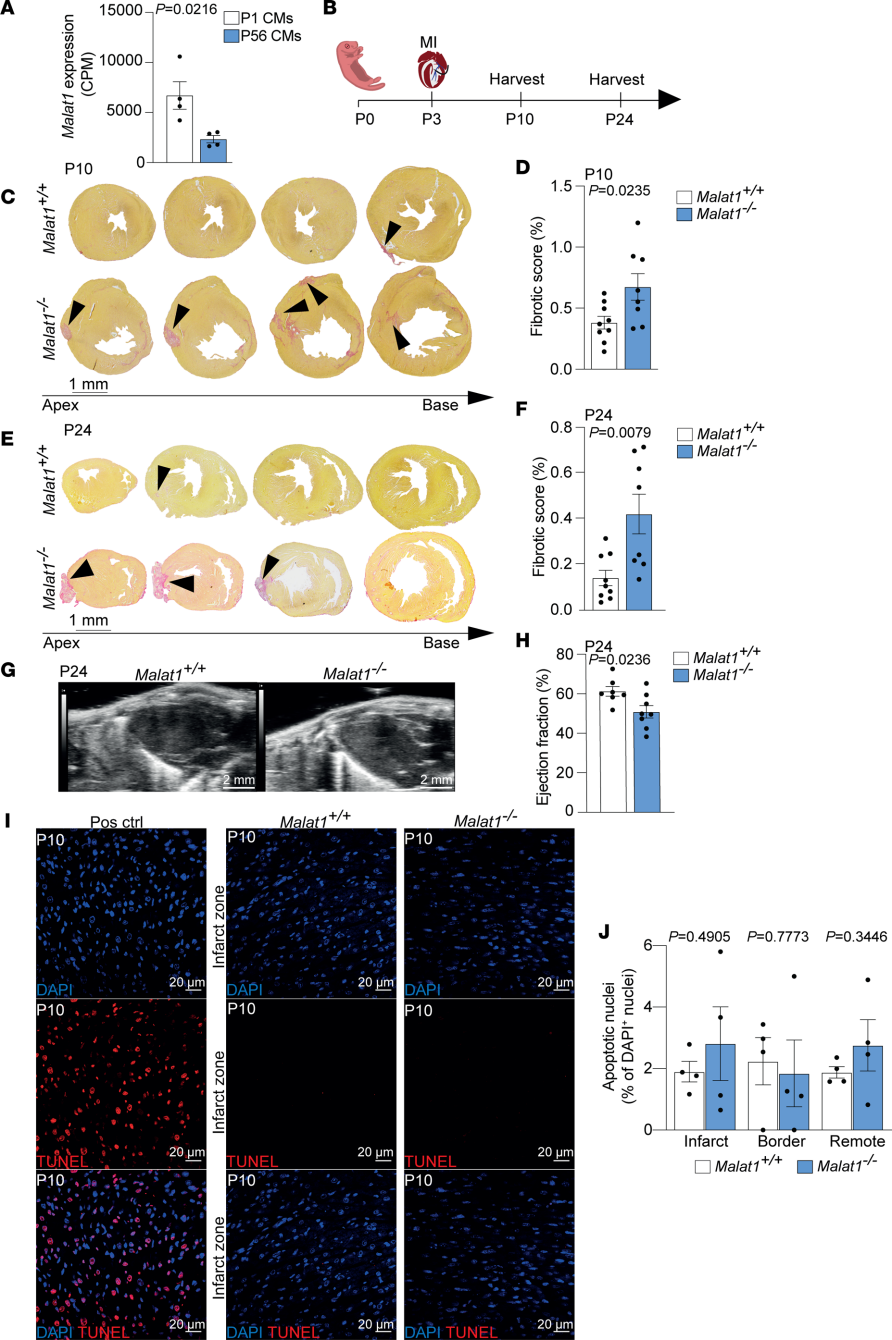
Malat1 deficiency prevents neonatal heart regeneration. (**A**) Expression level of *Malat1* (counts per million mapped reads, CPM) in regenerative (P1) versus mature nonregenerative cardiomyocytes (CMs) (P56). *n* = 4 for both groups. Available RNA-seq data were used for analysis (66). (**B**) Scheme of MI experiment in neonatal mice. (**C** and **D**) Sirius red staining of heart sections from *Malat1^+/+^* and *Malat1^–/–^* mice 7 days after MI. (**C**) Representative histology images of Sirius red staining. Panels show different cross sections from apex to base. Arrowheads indicate persistent scarring. (**D**) Quantification of scarring after MI. *n* = 9 for *Malat1^+/+^* and *n* = 8 for *Malat1^–/–^*. (**E** and **F**) Sirius red staining of heart sections from *Malat1^+/+^* and *Malat1^–/–^* mice 21 days after MI. (**E**) Representative histology images of Sirius red staining. Arrowheads indicate persistent scarring. (**F**) Quantification of scarring. *n* = 9 for *Malat1^+/+^* and *n* = 8 for *Malat1^–/–^*. (**G** and **H**) Echocardiographic analysis 21 days after MI. (**G**) Representative images of hearts in parasternal long-axis. (**H**) Quantification of the left ventricular ejection fraction. *n* = 7 for *Malat1^+/+^* and *n* = 8 for *Malat1^–/–^*. (**I** and **J**) Apoptosis analysis of *Malat1^+/+^* and *Malat1^–/–^* hearts 7 days after MI. (**I**) Representative images showing colocalization of TUNEL and DAPI in *Malat1^+/+^* and *Malat1^–/–^* hearts. (**J**) Quantification of TUNEL-positive cells in infarct, border, and remote zones. *n* = 4 for *Malat1^+/+^* and *n* = 4 for *Malat1^–/–^*. Scale bars: 1 mm (**C** and **E**), 2 mm (**G**), and 20 μm (**I**). Data are shown as mean ± SEM. *P* values were calculated by unpaired, 2-tailed Student’s *t* test (**A**, **D**, **H**, and **J**) or Mann-Whitney *U* test (**F**).

To gain first insights into a putative role of Malat1 in cardiac regeneration, we analyzed Malat1 expression in postnatal regenerating cardiomyocytes compared with adult cardiomyocytes. This analysis shows that Malat1 expression was significantly reduced in adult mature nonregenerative cardiomyocytes on postnatal day 56 (P56) when compared with neonatal proliferative regenerative cardiomyocytes on P1 (Figure 1A). These data raised the hypothesis that reduced Malat1 expression in adult mice might contribute to the limited cardiac regenerative capacity.

To test this hypothesis, we further studied the role of Malat1 in heart regeneration by inducing MI in 3-day-old (P3) *Malat1^–/–^* and *Malat1^+/+^* neonates by surgical occlusion of the left anterior descending (LAD) artery (33) (Figure 1B). Although neonatal mice are able to regenerate the myocardium 3 weeks after MI, we still detected some degree of scarring around the injury site. This finding is consistent with previous studies (1, 2). Malat1 deficiency significantly increased scar size 1 week and 3 weeks after MI. The heart of *Malat1^–/–^* mice showed higher fibrotic areas than the control littermates, both 1 week after MI (Figure 1, C and D) and 3 weeks after MI (Figure 1, E and F). Likewise, *Malat1^–/–^* mice showed impaired cardiac function, as assessed by significantly decreased ejection fraction 21 days after MI when compared with *Malat1^+/+^* mice (Figure 1, G and H, and Supplemental Videos 1 and 2; supplemental material available online with this article; https://doi.org/10.1172/jci.insight.162124DS1). These observations indicate that Malat1 is required for neonatal cardiac regeneration. Interestingly, we did not detect an increase in cell death in the hearts of *Malat1^–/–^* mice 1 week after MI (Figure 1, I and J).

**Figure 2:**
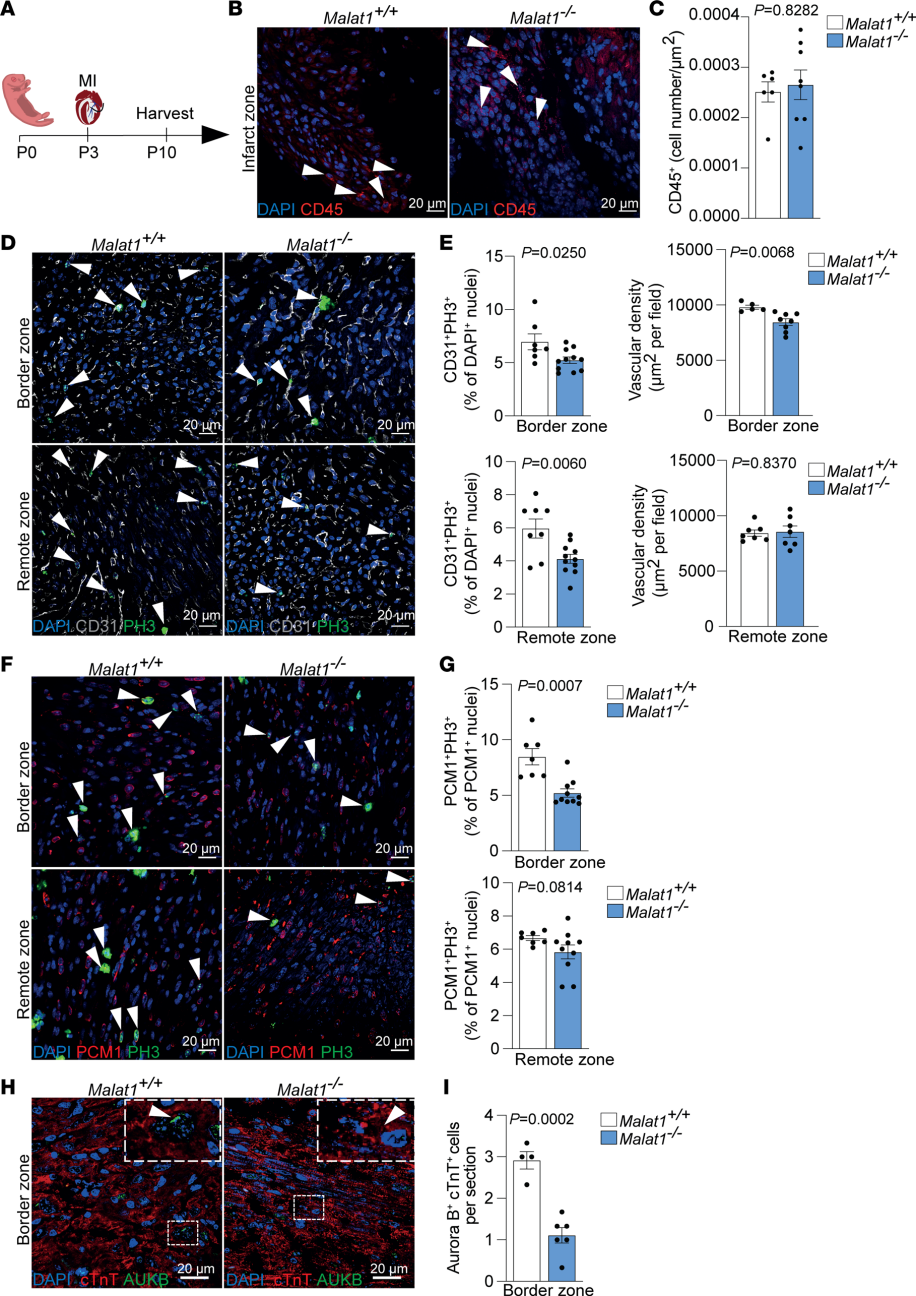
Malat1 deficiency impairs regenerative response and inhibits cardiomyocyte proliferation. (**A**) Schematic of MI experiment in neonatal mice. (**B** and **C**) CD45^+^ cell infiltration in infarct zone 7 days after MI. (**B**) Representative immunofluorescence images showing CD45^+^ cell infiltration in the infarct zone. Arrowheads indicate CD45^+^ cells. (**C**) Quantification of infiltrated CD45^+^ cells. *n* = 6 for *Malat1^+/+^* and *n* = 8 for *Malat1^–/–^*. (**D** and **E**) Vascular density and endothelial cell proliferation analysis 7 days after MI. (**D**) Representative immunofluorescence images of CD31^+^PH3^+^ cells from border (top) and remote (bottom) zones. Arrowheads indicate PH3^+^ endothelial cells. (**E**) Top: Quantification of CD31^+^ cell density (left) in the border zone. *n* = 5 for *Malat1^+/+^* and *n* = 8 for *Malat1^–/–^*. Quantification of CD31^+^PH3^+^ cells (right) in border zone. *n* = 7 for *Malat1^+/+^* and *n* = 11 for *Malat1^–/–^*. Bottom: Quantification of CD31^+^ cells density (left) in the remote zone. *n* = 7 for *Malat1^+/+^* and *n* = 7 for *Malat1^–/–^*. Quantification of CD31^+^PH3^+^ cells (right) in the remote zone. *n* = 8 for *Malat1^+/+^* and *n* = 11 for *Malat1^–/–^*. (**F** and **G**) Cardiomyocyte mitosis 7 days after MI. (**F**) Representative immunofluorescence images showing PH3^+^PCM1^+^ cells (mitotic cardiomyocytes) in border (top) and remote (bottom) zones. Arrowheads indicate PH3^+^PCM1^+^ cells. (**G**) Top: Quantification of PH3^+^PCM1^+^ cells in border zones. Bottom: Quantification of PH3^+^PCM1^+^ cells in remote zones. *n* = 7 for *Malat1^+/+^* and *n* = 10 for *Malat1^–/–^*. (**H** and **I**) Cardiomyocyte cytokinesis 7 days after MI. (**H**) Representative immunofluorescence images showing Aurora B^+^cTnT^+^ cells (dividing cardiomyocytes) in the border zone. (**I**) Quantification of Aurora B^+^cTnT^+^ cells in the border zone. *n* = 4 for *Malat1^+/+^* and *n* = 6 for *Malat1^–/–^*. Scale bars: 20 μm. All data are shown as mean ± SEM. *P* values were calculated by Mann-Whitney *U* test (**C** and **G**, top), unpaired, 2-tailed Student’s *t* test (**E** and **I**), or unpaired, 2-tailed Student’s *t* test with Welch’s correction (**G**, bottom).

To better understand the reduced regenerative capacity of *Malat1^–/–^* hearts, we characterized the cardiac phenotype 1 week after MI (Figure 2A). Although Malat1 deficiency has been linked to enhanced CD45^+^ cell infiltration in atherosclerotic lesions (28), *Malat1^–/–^* mice showed levels of CD45^+^ cells in the myocardium that were similar to those of control littermates (Figure 2, B and C). Furthermore, Malat1 is known to regulate endothelial cell function and vessel growth (27). Analysis of the microcirculation, both in the border and remote areas of the myocardium 1 week after MI, revealed reduced endothelial cell proliferation both in the border and remote zones (Figure 2, D and E), while vascular density was only reduced in the border zone (Figure 2, D and E). Since cardiomyocyte proliferation is indispensable for heart regeneration in neonatal mice (1), we additionally studied the effect of Malat1 deficiency on cardiomyocyte proliferation. Histological analysis revealed a reduction in the number of phospho-histone H3–positive (PH3-positive) cardiomyocytes in the border zone of the myocardium after MI and no differences in the remote zone (Figure 2, F and G). Of note, PH3 immunostaining is not sufficient to distinguish bona fide cardiomyocyte division from binucleation (34). To overcome this issue, we analyzed Aurora B–positive events in cardiomyocyte midbodies. Positioning of the Aurora B–positive midbody is a marker of a dividing cardiomyocyte. *Malat1^–/–^* hearts showed a reduction in Aurora B events in cardiomyocytes in the border zone of the myocardium after MI (Figure 2, H and I). Together, these observations show impaired proliferation in cardiomyocytes and endothelial cells in the heart of *Malat1^–/–^* mice after MI.

### Malat1 deficiency increases cardiomyocyte binucleation.

**Figure 3:**
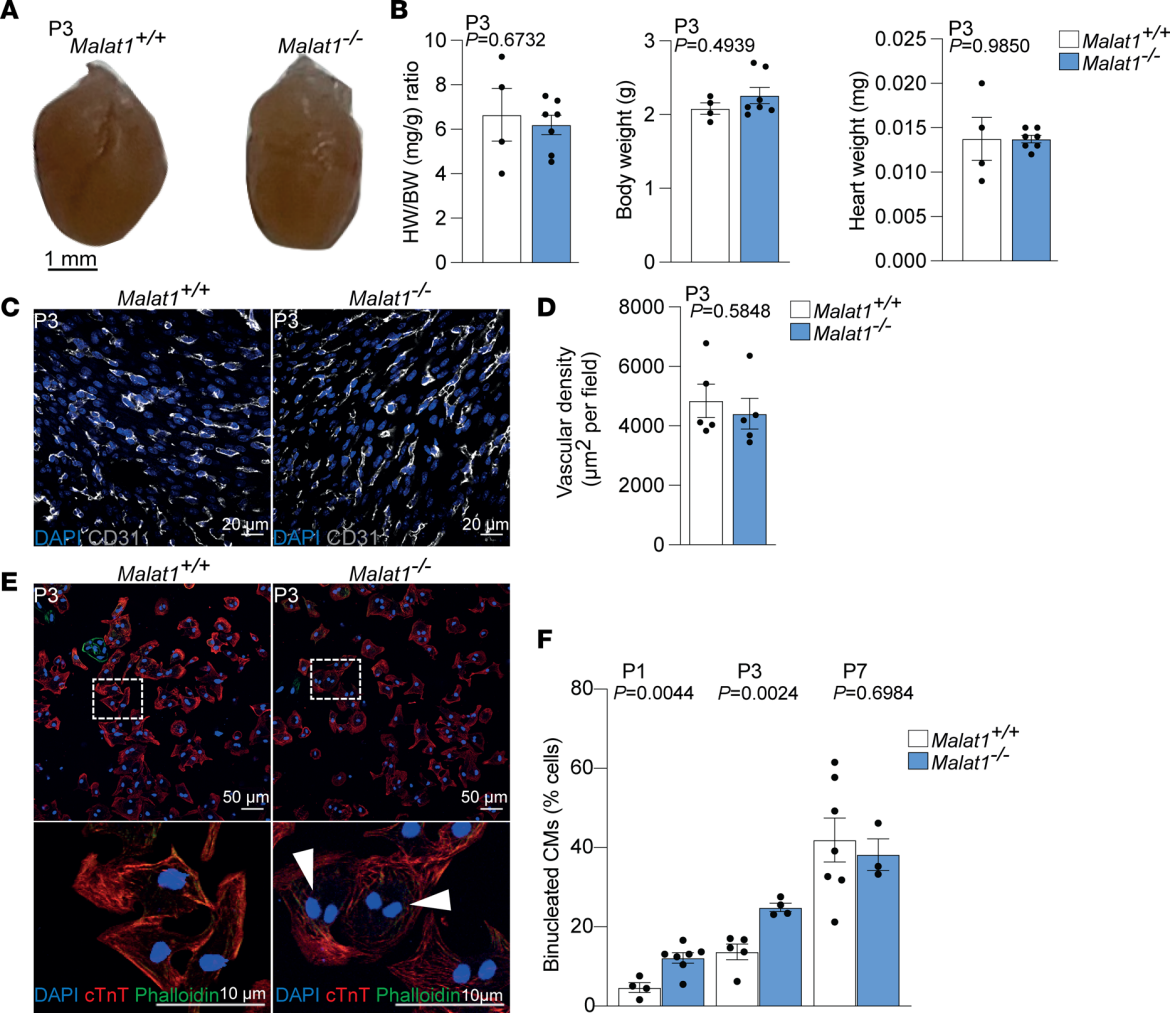
*Malat1^–/–^* mice show increased number of binucleated cardiomyocytes on P3. (**A**) Macroscopic analysis of hearts from *Malat1^+/+^* and *Malat1^–/–^* mice on P3. (**B**) Heart weight (HW) to body weight (BW) ratio, body weight, and heart weight comparisons. *n* *=* 4 for *Malat1^+/+^* and *n* = 7 for *Malat1^–/–^*. (**C** and **D**) Vascular density analysis of *Malat1^+/+^* and *Malat1^–/–^* hearts on P3. (**C**) Representative immunofluorescence images of CD31^+^ cells. (**D**) Quantification of CD31^+^ cell density. *n* = 5 for *Malat1^+/+^* and *n* = 5 for *Malat1^–/–^*. (**E** and **F**) Analysis of cardiomyocyte (CM) binucleation on P1, P3, and P7. (**E**) Representative immunofluorescence images of mono- and binucleated cardiomyocytes on P3. (**F**) Quantification of binucleated cardiomyocytes on P1, P3, and P7. *n* *=* 4 for *Malat1^+/+^* and *n* = 7 for *Malat1^–/–^* (P1), *n* = 5 for *Malat1^+/+^* and *n* = 4 for *Malat1^–/–^* (P3), *n* = 7 for *Malat1^+/+^* and *n* = 3 for *Malat1^–/–^* (P7). Scale bars: 20 μm (**C**) and 50 μm (**E**). Data are shown as mean ± SEM. *P* values were calculated by unpaired, 2-tailed Student’s *t* test between each group for each time point.

In order to understand whether the deletion of Malat1 has an effect on the heart at baseline that could inhibit regeneration, we studied the hearts on P3, the same day when we had previously induced MI in the mice. We did not detect obvious macroscopic differences between mutant and control hearts (Figure 3A); heart weight, body weight, and the heart weight to body weight ratio were not affected (Figure 3B), and vascular density was not altered at this stage (Figure 3, C and D). Analysis of isolated cardiomyocytes, however, revealed an increase in the percentage of binucleated cardiomyocytes in *Malat1^–/–^* hearts on P1 and P3 (Figure 3, E and F). Interestingly, the difference between *Malat1^–/–^* and wild-type mice disappeared on P7 when approximately 40% of cardiomyocytes also acquired a binucleated state in wild-type mice (Figure 3, E and F). Cardiomyocyte polyploidy and binucleation have been shown to prevent heart regeneration in zebrafish (35). In mice, the transition from mononucleated to binucleated cardiomyocytes occurs after the first week of life when neonatal mice lose their regenerative capacity (8, 36) and variability in binucleation of different mouse strains underlies natural variation in heart regeneration (37). Therefore, our findings may suggest that Malat1 deficiency accelerates cardiomyocyte binucleation, and thus, alters the regeneration window of the neonatal heart, rendering *Malat1^–/–^* neonatal hearts nonregenerative.

**Figure 4:**
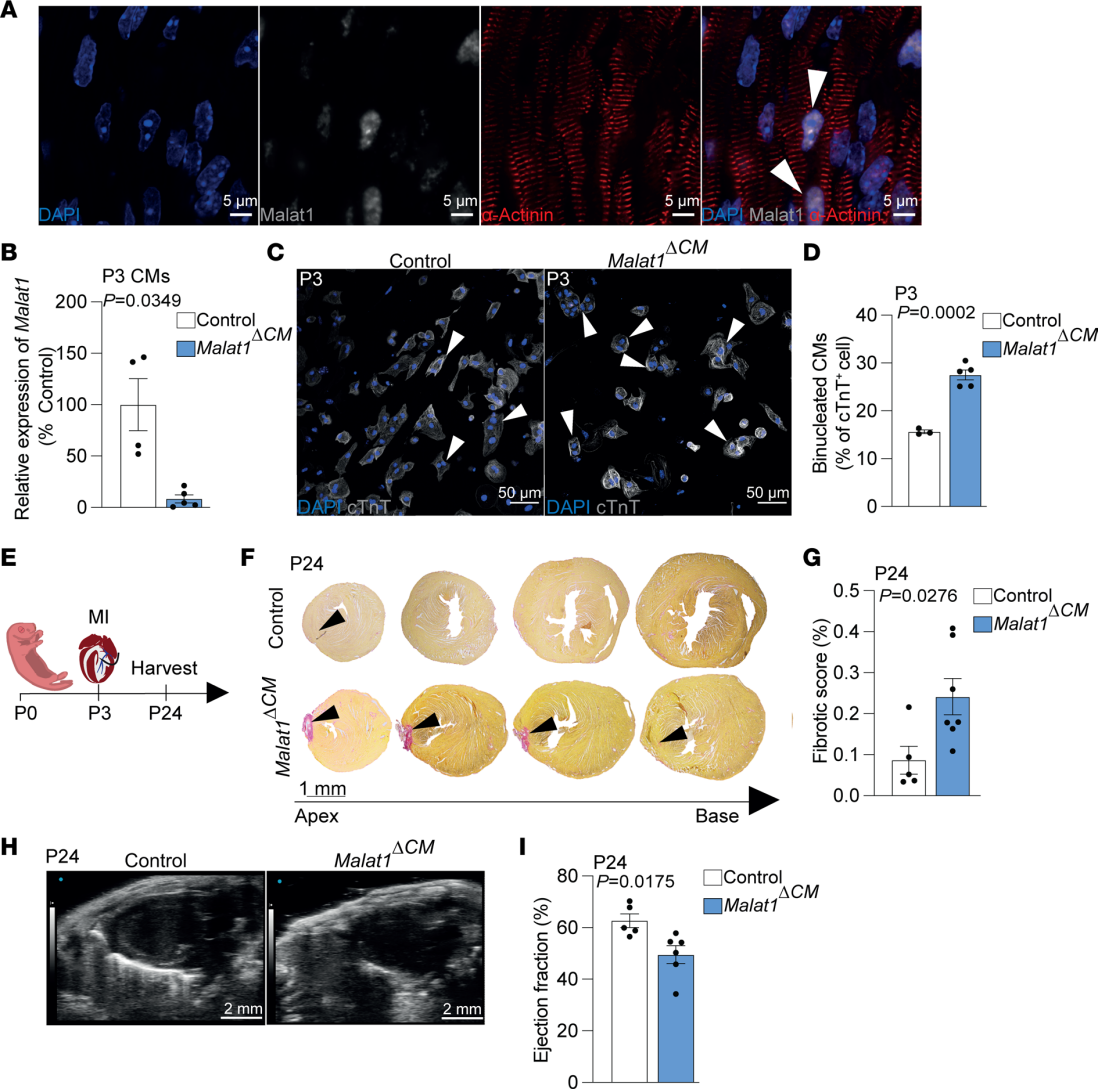
Cardiomyocyte-specific deletion of Malat1 abolishes cardiac regeneration and induces cardiomyocyte binucleation. (**A**) Representative images of RNAscope in situ hybridization. Arrowheads indicate *Malat1* transcripts in neonatal cardiomyocytes (α-Actinin^+^). (**B**) RT-qPCR–based detection of *Malat1* levels in control and *Malat1^ΔCM^* cardiomyocytes (CMs) on P3. (**C**) Representative immunofluorescence images of isolated cardiomyocytes (cTnT^+^ cells) from control and *Malat1^ΔCM^* mice on P3. Arrowheads indicate binucleated cardiomyocytes. (**D**) Quantification of binucleated cardiomyocytes on P3. *n* = 3 for control and *n* = 5 for *Malat1^ΔCM^*. (**E**) Schematic of MI experiment in neonatal mice. (**F**) Representative histology images of Sirius red staining 21 days after MI. Arrowheads indicate persistent scarring. (**G**) Quantification of scarring. *n* = 5 for control and *n* = 7 for *Malat1^ΔCM^*. (**H**) Representative images of echocardiography analysis of the hearts in parasternal long axis. (**I**) Quantification of the left ventricular ejection fraction. *n* = 5 for control and *n* = 6 for *Malat1^ΔCM^*. Scale bars: 5 μm (**A**), 50 μm (**C**), and 2 mm (**H**). Data are shown as mean ± SEM. *P* values were calculated by unpaired, 2-tailed Student’s *t* test with Welch’s correction (**B**) or unpaired, 2-tailed Student’s *t* test (**D**, **G**, and **I**).

**Figure 5:**
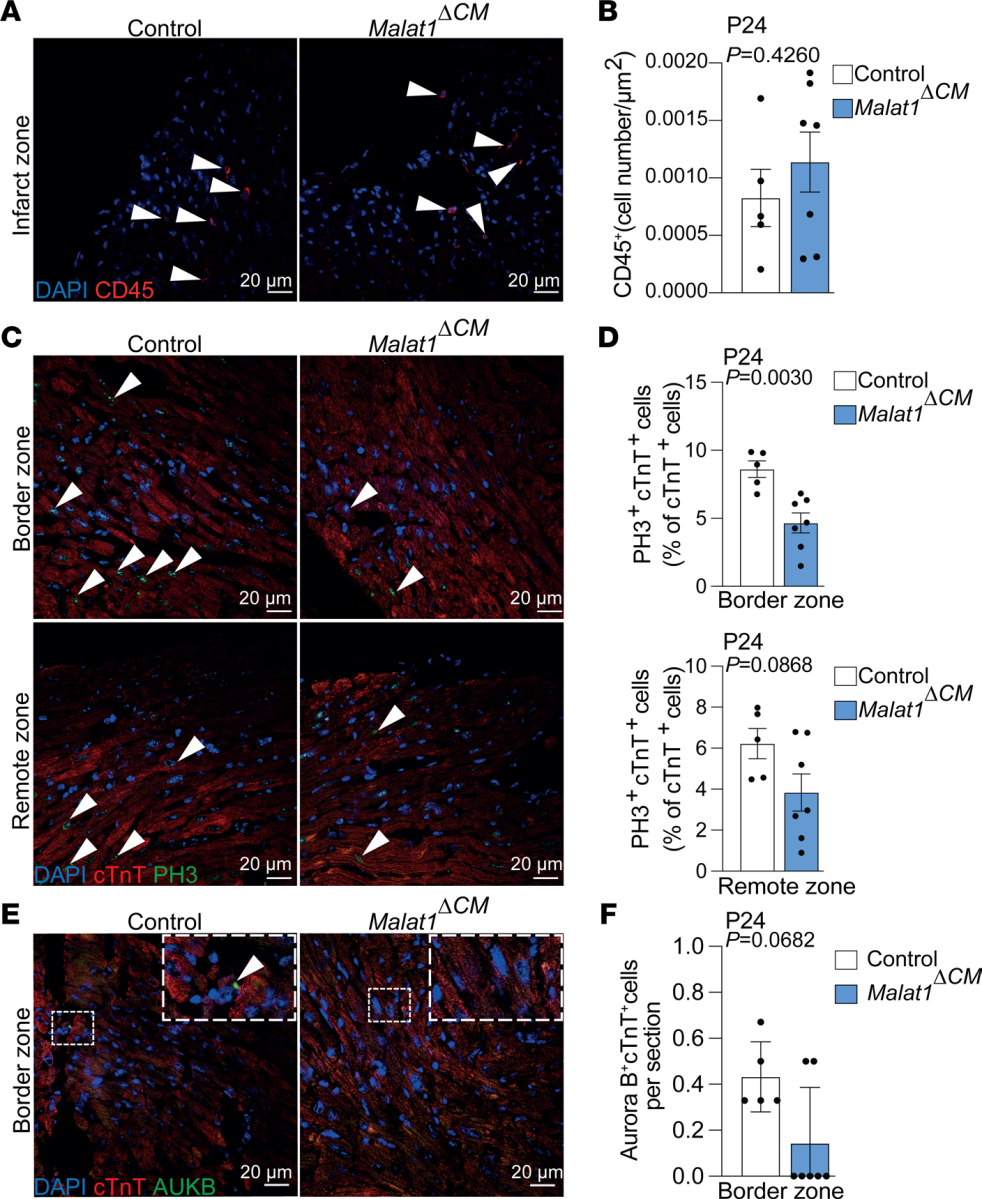
Cardiomyocyte-specific deletion of Malat1 reduced cardiomyocyte proliferation 21 days after MI. (**A** and **B**) CD45^+^ cell infiltration in infarct zone 21 days after MI. (**A**) Representative immunofluorescence images showing CD45^+^ cell infiltration in the infarct zone. Arrowheads indicate CD45^+^ cells. (**B**) Quantification of infiltrated CD45^+^ cells. *n* = 5 for Control and *n* = 7 for *Malat1^ΔCM^*. (**C** and **D**) Cardiomyocyte mitosis 21 days after MI. (**C**) Representative immunofluorescence images showing PH3^+^cTnT^+^ cells (mitotic cardiomyocytes) in border (top) and remote (bottom) zones. Arrowheads indicate PH3^+^cTnT^+^ cells. (**D**) Top: Quantification of PH3^+^cTnT^+^ cells in border zones. Bottom: Quantification of PCM1^+^PH3^+^ cells in remote zones. *n* = 5 for Control and *n* = 7 for *Malat1^ΔCM^*. (**E** and **F**) Cardiomyocyte cytokinesis 7 days after MI. (**E**) Representative immunofluorescence images showing Aurora B^+^cTnT^+^ cells (dividing cardiomyocytes) in the border zone. (**F**) Quantification of Aurora B^+^cTnT^+^ cells in the border zone. *n* = 5 for Control and *n* = 7 for *Malat1^ΔCM^*. Scale bars: 20 μm. Data are shown as mean ± SEM. *P* values were calculated by unpaired, 2-tailed Student’s *t* test (**B** and **D**) or Mann-Whitney *U* test (**F**).

Malat1 is highly expressed in cardiomyocytes from neonatal hearts (Figures 1A and 4A). To test our hypothesis that the cardiomyocyte-specific loss of Malat1 induces cardiomyocyte binucleation and hinders regeneration, we deleted Malat1 in cardiomyocytes and induced MI in a similar manner as we have done with *Malat1^–/–^* mice. To do so, we bred animals bearing a conditional *Malat1* loss-of-function allele (38) with *Myh6-Cre* transgenic animals that express Cre recombinase especially in cardiomyocytes (39). *Malat1*^ΔCM^ mice showed significantly reduced expression of Malat1 in isolated cardiomyocytes on P3 (Figure 4B). First, we tested whether Malat1 deletion in cardiomyocytes was sufficient to induce increased binucleation on P3, as we had observed in the *Malat1^–/–^* mice. Indeed, *Malat1*^ΔCM^ mice showed a significant increase in the number of binucleated cardiomyocytes at this stage (Figure 4, C and D). Then, we induced MI in *Malat1*^ΔCM^ mice and control littermates 3 days after birth and analyzed the resulting scar 3 weeks after the intervention (Figure 4E). *Malat1*^ΔCM^ mice showed reduced capacity to regenerate, as the resulting scar after the ischemic damage was significantly larger than in the control littermates (Figure 4, F and G). Moreover, echocardiography analysis revealed that *Malat1*^ΔCM^ mice show impaired left ventricular ejection fraction (Figure 4, H and I, and Supplemental Videos 3 and 4). Similar to our observations in global *Malat1*-knockout mice, cardiomyocyte-specific deletion of *Malat1* did not affect the infiltration of CD45^+^ cells after MI (Figure 5, A and B). Furthermore, we observed a reduction in cardiomyocyte proliferation in *Malat1*^ΔCM^ mice after MI. In detail, *Malat1*^ΔCM^ mice showed reduced numbers of PH3–positive cardiomyocytes in the border zone of the myocardium but no significant differences in the remote zone (Figure 5, C and D). Furthermore, we observed a 67.5% reduction in the number of Aurora B–positive events in the border zone of *Malat1*^ΔCM^ myocardium after MI (*P* = 0.06) (Figure 5, E and F). Together, these results confirm our hypothesis that the impeded regeneration observed in *Malat1^–/–^* mice is caused by a cell-autonomous effect of Malat1 in cardiomyocytes.

**Figure 6:**
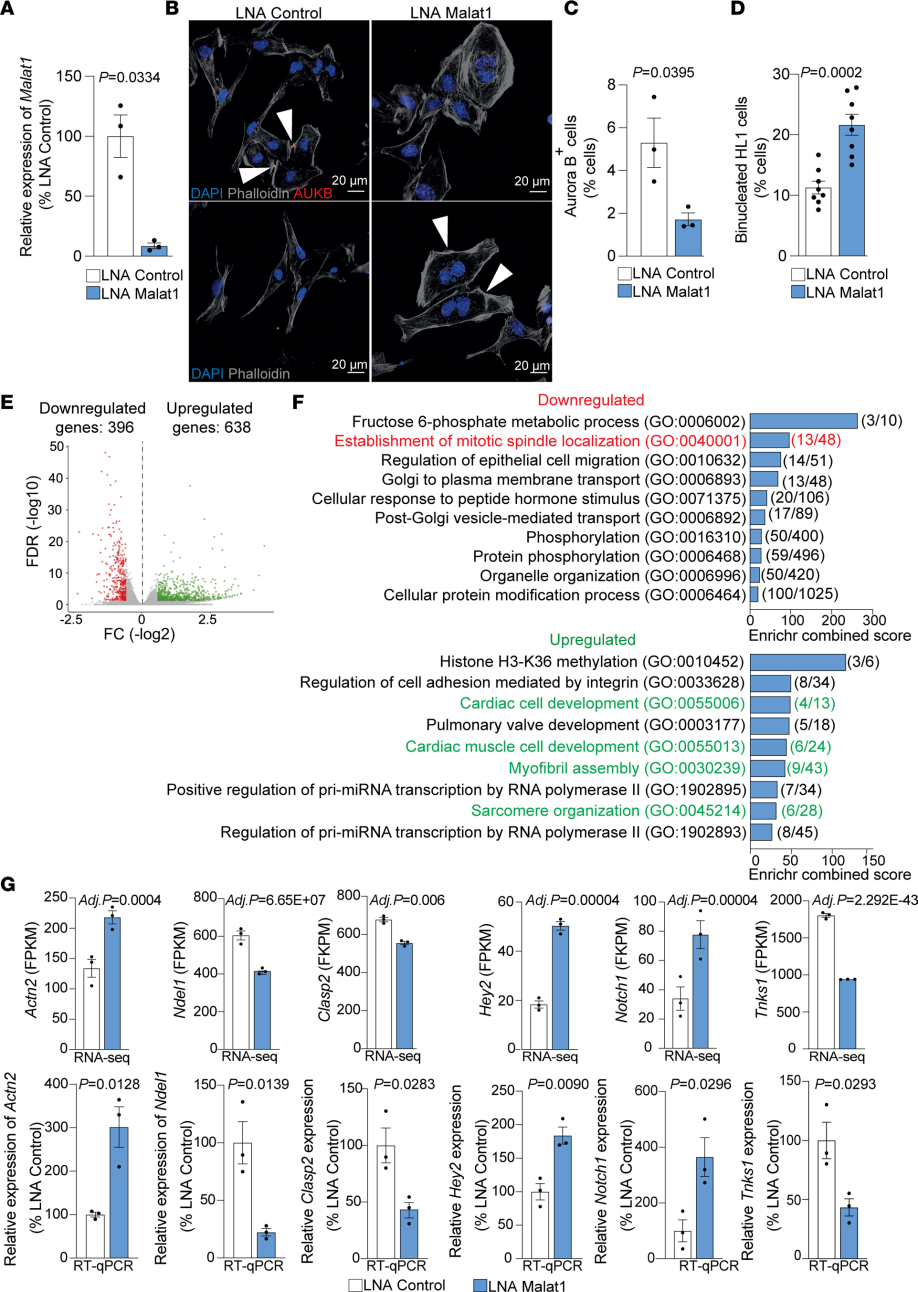
LNA-mediated silencing of Malat1 induces binucleation in mouse HL-1 cells and regulates cardiac maturation and mitosis-associated gene expression. (**A**) RT-qPCR–based confirmation of *Malat1* silencing in mouse HL-1 cells. *n* = 3. (**B**–**D**) Proliferation and binucleation analysis after *Malat1* silencing in mouse HL-1 cells. (**B**) Representative immunofluorescence images of binucleated (upper figures) or proliferating cells (lower figures, Aurora B^+^ cells). Scale bars: 20 μm. (**C**) Quantification of Aurora B^+^ mouse HL-1 cells (proliferating cells) 48 hours after *Malat1* silencing. *n* = 3. (**D**) Quantification of binucleated mouse HL-1 cells 72 hours after *Malat1* silencing. *n* = 8. (**E**) Volcano plot of down- and upregulated genes upon *Malat1* silencing. *P* < 0.05, log_2_(fold change) < –0.585 or > 0.585, FDR ≤ 0.5. (**F**) GO enrichment analysis of differentially expressed genes. Numbers in brackets indicate the number of genes regulated in the GO terms. (**G**) Representation of gene expression analysis of selected genes by RNA sequencing (upper row) and confirmation by RT-qPCR analysis (bottom row). *n* = *3*. Data are shown as mean ± SEM. *P* values were calculated by unpaired, 2-tailed Student’s *t* test with Welch’s correction (**B**) or unpaired, 2-tailed Student’s *t* test (**C**–**E**).

To validate the relationship between Malat1 and cardiomyocyte proliferation and binucleation, we studied the role of Malat1 in HL-1 cells in vitro. HL-1 cells are contractile cells that retain characteristics of adult cardiomyocytes in vitro (40). We depleted Malat1 in HL-1 cells using locked nucleic acid gapmers (LNA-gapmers) (Figure 6A) and then studied proliferation and binucleation. Aurora B immunostaining revealed a reduction in proliferation in HL-1 cells after Malat1 silencing (Figure 6, B and C). Furthermore, assessment of nuclei number confirmed the increased binucleation in these cardiomyocyte-like cells (Figure 6, B and D). To gain insight into the molecular processes occurring after silencing Malat1, we performed gene expression analysis of HL-1 cells treated with Malat1 LNA-gapmer (LNA Malat1) and control LNA-gapmer (LNA Control) by RNA sequencing. Differential gene expression analysis revealed 1034 differentially expressed genes (adjusted *P* value < 0.05): 638 upregulated and 396 downregulated (Figure 6E). Gene Ontology (GO) analysis of differentially expressed genes was performed using the Enrichr database (41, 42). GO analysis revealed an upregulation of genes related to cardiac cell development, cardiac muscle cell development, myofibril assembly, and sarcomere organization (Figure 6F), and downregulation of genes related to the establishment of mitotic spindle localization (Figure 6F). The expression of structural genes of the sarcomere like *Tnnt3*, *Ttn*, and *Myl2* and the expression of genes related to Z-disc maturation like *Actn2* and *Ankrd1* was upregulated in Malat1-deficient cells. This increase in gene expression is required for cardiomyocyte maturation (43). On the other hand, the expression of genes related to microtubule dynamics at the mitotic spindle like *Numa1*, *Ndel1*, *Tnks1,* *Clasp1*, and *Clasp2* was downregulated upon Malat1 knockdown (Figure 6G and Supplemental Figure 1). Numa1 and Tnks1 are regulated by Plk1 during mitosis and regulate spindle elongation (44, 45). Numa1 recruits the dynein-dynactin complex directly to the cell membrane (45). Furthermore, Ndel1 establishes the organization of microtubule arrays at the centrosome (46). Throughout mitosis, Clasp1 and Clasp2 play a crucial role in ensuring bipolar alignment of chromosomes on the mitotic spindle (47, 48).

Furthermore, we found it of interest that the gene expression of *Notch1* and *Hey2*, crucial members of the Notch signaling pathway, is upregulated upon Malat1 silencing (Figure 6G). The Notch signaling pathway is well documented to play an important role in controlling heart development and maturation (49–51). This suggests that Malat1 deficiency not only reduces proliferation and induces binucleation, but also leads to the activation of a maturation gene expression program in cardiomyocytes.

### Malat1 interacts with hnRNP U.

Malat1 has been shown to control the cell cycle in tumor cells by interacting with heterogeneous nuclear ribonucleoprotein C (hnRNP C) (52). To study whether Malat1 controls cardiomyocyte proliferation and binucleation in a similar manner, we silenced hnRNP C expression in HL-1 cells using siRNAs (Supplemental Figure 2A). However, this did not induce binucleation in this cardiomyocyte cell line (Supplemental Figure 2, B and C).

**Figure 7:**
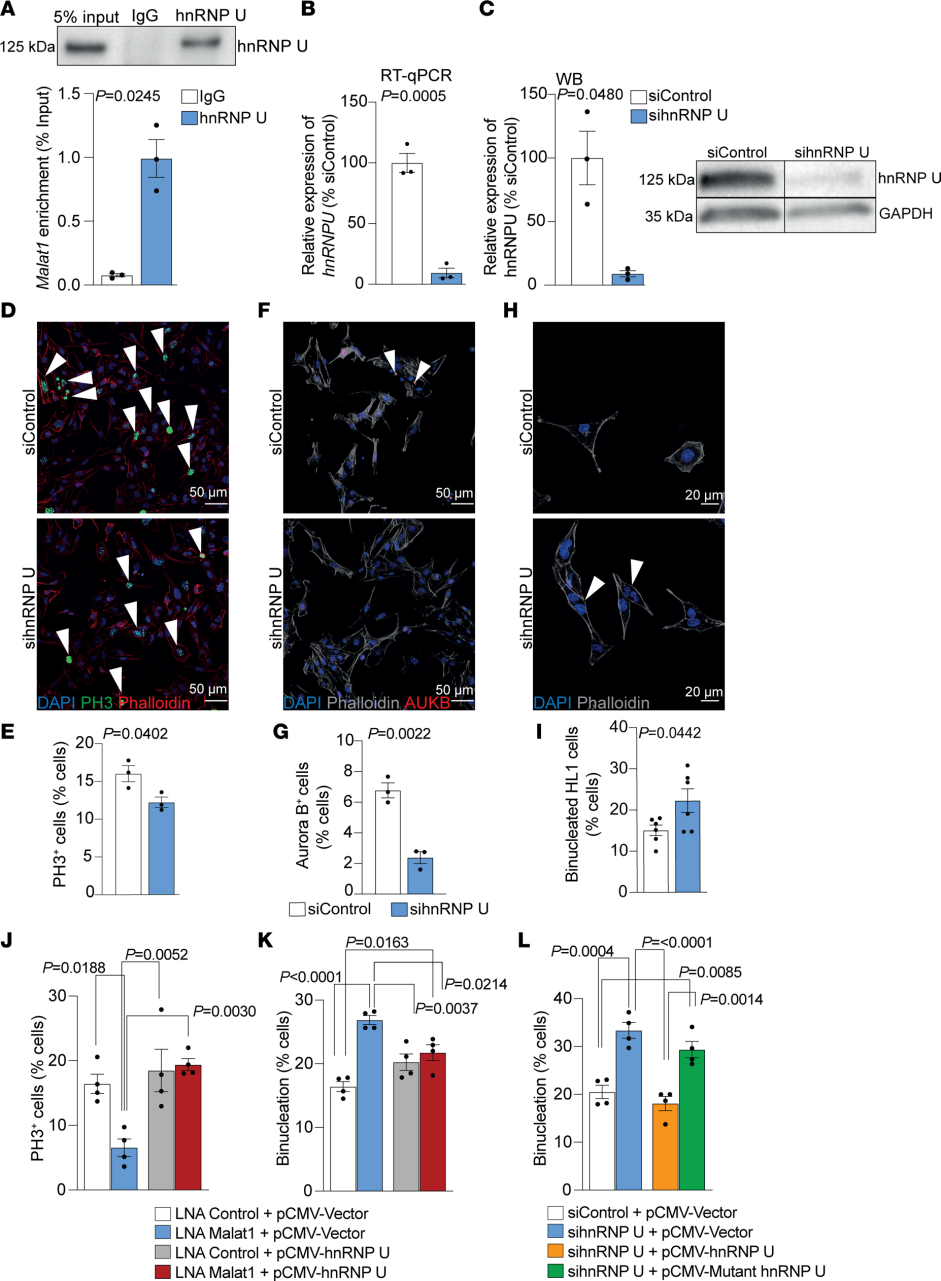
Malat1 interacts with hnRNP U and hnRNP U deficiency induces binucleation in HL-1 cells. (**A**) Enrichment of *Malat1* in anti–hnRNP U immunoprecipitations. *n* =3. (**B** and **C**) RT-qPCR– and Western blot–based confirmation of hnRNP U silencing in HL-1 cells. Representative image of Western blot is shown. Lanes were run on the same gel but were noncontiguous; the complete Western blot is shown in Supplemental Figure 3F. *n* = 3. (**D** and **E**) Analysis of HL-1 cell proliferation 48 hours after hnRNP U knockdown. (**D**) Representative immunofluorescence images of PH3^+^ (mitotic) cells. Arrowheads indicate mitotic HL-1 cells. (**E**) Quantification of PH3^+^ HL-1 cells. *n* = 3. (**F** and **G**) Cytokinesis of HL-1 cells 48 hours after hnRNP U knockdown. (**F**) Representative immunofluorescence images of Aurora B^+^ (proliferating) HL-1 cells. Arrowheads show proliferating HL-1 cells. (**G**) Quantification of Aurora B^+^ HL-1 cells *n* = 3. (**H** and **I**) Binucleation analysis of HL-1 cells 72 hours after hnRNP U knockdown. (**H**) Representative immunofluorescence images of binucleated HL-1 cells. Arrowheads indicate binucleated cells. (**I**) Quantification of binucleated HL-1 cells. *n* = 6. (**J**) Quantification of PH3^+^ HL-1 cells after Malat1 knockdown and hnRNP U overexpression. *n* = 4. (**K**) Quantification of binucleated HL-1 cells after Malat1 knockdown and hnRNP U overexpression. *n* = 4. (**L**) Quantification of binucleated HL-1 cells after wild-type and mutant hnRNP U overexpression. *n* = 4. Scale bars: 50 μm (**D** and **F**) and 20 μm (**H**). Data are shown as mean ± SEM. *P* values were calculated by unpaired, 2-tailed Student’s *t* test with Welch’s correction (**A** and **C**), Student’s *t* test (**B** and **D**–**I**), or 1-way ANOVA with Tukey’s multiple-comparison test (**J–L**).

However, RNA immunoprecipitation assays revealed that Malat1 interacts with hnRNP U (Figure 7A). hnRNP U is known to be an important regulator of mitosis (53, 54) and it has been already linked with postnatal heart development and function (55). Furthermore, *Hnrnpu*-mutant mice develop a lethal dilated cardiomyopathy phenotype (55). To study whether hnRNP U might play a role in the regulation of cardiomyocyte proliferation and binucleation, we silenced it in HL-1 cells using siRNAs (Figure 7, B and C). Similar to what we have observed in Malat1-deficient HL-1 cells, hnRNP U–silenced cells showed reduced cellular proliferation. In addition, we detected fewer PH3-positive cells (Figure 7, D and E) and fewer Aurora B–positive midbody events (Figure 7, F and G). Finally, hnRNP U deficiency also induced binucleation in HL-1 cells (Figure 7, H and I). To determine whether excess hnRNP U can rescue the Malat1 deficiency–induced decrease in cardiomyocyte proliferation, we overexpressed hnRNP U in Malat1-silenced HL-1 cells (Supplemental Figure 3A). Indeed, overexpression of hnRNP U was sufficient to rescue the reduced proliferation in Malat1-deficient HL-1 cells (Figure 7J and Supplemental Figure 3C) and prevented the coinciding binucleation (Figure 7K and Supplemental Figure 3C). These data suggest that excessive hnRNP U can compensate for proliferation defects induced by Malat1 deficiency.

To finally determine whether hnRNP U mediates its effect on binucleation via its RNA binding activity, we tested the effect of an hnRNP U mutant that lacks its RNA binding motif (Supplemental Figure 3B). In contrast to hnRNP U wild-type protein, which rescues the increased binucleation induced by silencing of hnRNP U, no rescue was detected when overexpressing the mutant hnRNP U, which lacks the RNA binding motif (Figure 7L and Supplemental Figure 3D). Furthermore, the mutant version of hnRNP U did not rescue the binucleation induced by Malat1 deficiency in HL-1 cells (Supplemental Figure 3E). Together, these observations suggest that Malat1 might control cell cycle progression and cytokinesis in cardiomyocytes via hnRNP U.

## Discussion

Here, we show for the first time to our knowledge that the lncRNA Malat1 controls heart regeneration. We demonstrate that Malat1 is required for cardiomyocytes to remain proliferative and mononucleated. In the mouse, cardiomyocytes undergo a transition from a mononucleated to a binucleated state in the first week of life (8, 36). This transition accompanies the loss of regenerative potential in the mouse myocardium (1, 2) and has been considered to be causative for the loss (9). Zebrafish have a remarkably high capacity for regenerating their myocardium (56) and this is mainly achieved by the proliferation of existing cardiomyocytes (3, 57). Similar to our observation, induced binucleation or polyploidy in zebrafish has been shown to be sufficient to block regeneration (35). Malat1 knockdown induces this mature state in cardiomyocytes as early as 1 day after birth, altering the time window when mice are able to regenerate. Interestingly, we have also observed the upregulation of genes associated with cardiomyocyte maturation in Malat1-deficient HL-1 cells. Thus, genes like *Tnnt3*, *Ttn*, *Myl2*, *Actn2*, *Ankrd1*, and Notch signaling pathway genes like *Notch1* and *Hey2* were upregulated in these cells when treated with an LNA against Malat1. Furthermore, Malat1 silencing induced the downregulation of genes related to the establishment of mitotic spindle localization like *Numa1*, *Tnks1*, *Clasp1*, and *Clasp2*. The mitotic spindle is part of the cytokinesis machinery and its inhibition can lead to abnormal cytokinesis and binucleation (58).

In addition to the demonstration of reduced cardiac regeneration, we detected a slightly larger fibrotic area in the global *Malat1*–knockout mice when compared with the cardiomyocyte-specific *Malat1*-deficient mice, suggesting that Malat1 may additionally control other reparative processes in noncardiomyocytes. While we ruled out effects on inflammatory cell invasion, we indeed found reduced vascularization in *Malat1^–/–^* mice. This is consistent with previous reports by us and others showing that Malat1 is a crucial regulator of endothelial cell proliferation and migration (27, 59) and is required for ischemia-induced neovascularization in the hind limb (27). Deletion of Malat1 may thus affect both cardiomyocyte intrinsic and vascular repair mechanisms, which together promote regeneration.

We showed that Malat1 interacts with hnRNP U in cardiomyocytes. hnRNP U binds RNA by a low complexity RNA-binding RGG repeat and controls the operation of diverse protein and nucleoprotein machines. In the nucleus, hnRNP U binds to chromatin-associated RNAs and regulates chromatin structure (53, 54). Upon mitotic chromosome segregation, hnRNP U is phosphorylated by Aurora B and evicts RNA from prophase chromosomes (53). hnRNP U has also been shown to control pre-mRNA splicing during cardiac development and its deletion induced lethal cardiomyopathies (55). Here we show that deletion of hnRNP U also increases binucleation, suggesting that it is important for cytokinesis. Moreover, overexpression of hnRNP U did rescue reduced proliferation and prevented Malat1 deficiency–induced binucleation. Therefore, our data suggest that Malat1 interactions with hnRNP U might be required to mediate cardiomyocyte proliferation. Since adult cardiomyocytes can activate cell cycle progression but fail to efficiently divide, leading to binucleation or polyploidization, this process might be also important in attempts at cardiac regeneration of the adult heart.

## Methods

### Mouse strains.

*Malat1^–/–^ and Malat1^fl/fl^* mouse lines were described previously (38). Wild-type littermates were used as control for the *Malat1^–/–^* mice. Cardiomyocyte-specific deletion of *Malat1* was established by cross-breeding *Malat1^fl/fl^* mice with a *Myh6-Cre* line (39). *Malat1^fl/fl^;Myh6-Cre^+/+^* mice are referred to as control and *Malat1^fl/fl^;Myh6-Cre^Cre/+^* mice are referred to as *Malat1*^ΔCM^. Cardiomyocyte-specific deletion of *Malat1* was validated by RT-qPCR analysis from cardiomyocyte RNA. Mice were housed at 23°C ambient temperature and 60% humidity in 12-hour light/dark cycles.

### MI.

Neonatal mice on P3 were anesthetized by inducing hypothermia on ice, as described previously (2, 33). After transverse skin incision, lateral thoracotomy was applied at the fourth intercostal space by blunt dissection of intercostal muscles. To induce MI, an 8-0 prolene suture (Ethicon) was passed through the heart and was tied below the LAD coronary artery. Using a nonabsorbable 6-0 suture (Ethicon), the chest was stitched and skin glue was applied to join the skin together. Blood and remaining skin glue were removed with an alcohol solution. Pups were warmed under a heat lamp for several minutes until recovery and were placed back with their mother. Mice were euthanized 7 or 21 days after surgery.

### Echocardiography.

Echocardiography was performed 21 days after MI using a Vevo 3100 imaging system (VisualSonics) with the MX550 or MX700 probe. Parasternal long-axis views, short-axis views, and 2-dimensional-guided M-mode images of the short axis at the papillary muscle level were captured. To minimize variability between the measurements, all echocardiographic measurements were analyzed by VevoLab 3.2.5 software using artificial intelligence–based Auto-LV technology.

### Histology.

Postnatal hearts were perfused with PBS and incubated in 4% formaldehyde (Thermo Fisher Scientific, 28908) at 4°C overnight. Hearts were processed for paraffin embedding using standard procedures in the institute. Hearts were sectioned at 4 μm intervals and used for immunohistochemistry.

### Sirius red staining.

Slides were deparaffinized and rehydrated. Slides were stained in 0.1% Picro Sirius red solution (Waldeck and Sigma-Aldrich) according to the manufacturers’ instructions. Slides were mounted with Pertex mounting medium (Histolab, LEIC811). Images were acquired using an inverted light microscope (Nikon) with 2× objective. To determine the fibrotic score, ImageJ software (NIH, version 1.53a) was used to measure the positive fibrotic area and establish a ratio versus the total tissue area. At least 5 sections per animal were used for quantification.

### TUNEL staining.

TdT-mediated dUTP nick-end labeling (TUNEL) assays were conducted on paraffin-embedded heart sections. To this end, the In Situ Cell Death Detection Kit, Fluorescein (Roche, 11684795910) was used according to the manufacturer’s instructions. TUNEL-positive cells were analyzed in border, remote, and infarct zones.

### Immunohistochemistry.

Paraffin-embedded heart sections were deparaffinized and rehydrated by incubation in xylene followed by incubation in descending ethanol concentrations (100%, 80%, 70%, and 50% in water) and finally washed in PBS. For Aurora B staining, antigen retrieval was additionally performed. To do so, slides were boiled for 15 minutes in 10 mM citrate buffer, pH 6.0. Slides were cooled down and washed 3 times with PBS. Afterwards, sections were permeabilized with 0.1% Triton X-100 in PBS for 15 minutes at room temperature, and were blocked in 2% BSA and 1% donkey serum for 1 hour at room temperature. Slides were stained with primary antibodies overnight at 4°C in a humid chamber and washed 3 times with PBS, followed by incubation with secondary antibodies for 1 hour at room temperature. Slides were mounted using Fluoromount-G mounting medium (Thermo Fisher Scientific, 00495802). Immunostainings were imaged using a Zeiss LSM780 confocal microscope and analyzed using Volocity software 6.5.1 (Quorum Technologies).

The following primary and secondary antibodies were used: rabbit anti–p-histone H3 (EMD, 06570; 1:100), mouse anti-PCM1 (Santa Cruz Biotechnology, sc398365; 1:50), rat anti-CD31 (Dianova, DIA-310; 1:30), rabbit anti–Aurora B (Abcam, ab2254; 1:500), mouse anti–troponin T (Thermo Fisher Scientific, MS295; 1:100), mouse anti-CD45 (Abcam, ab8216; 1:100), donkey anti-mouse–Alexa Fluor 555 (Invitrogen, A-31570; 1:200), donkey anti-rat–Alexa Fluor 647 (Invitrogen, A48272; 1:200), and donkey anti-rabbit–Alexa Fluor 488 (Invitrogen, A21206; 1:200).

### Isolation of neonatal mouse cardiomyocytes.

Cardiomyocytes were isolated from P1, P3, and P7 pups. Neonatal hearts were dissected, atria were cut off, and ventricles were collected in ice-cold 20 mM 2,3-butanedione 2-monoxime (BDM) solution in HBSS. Ventricles were then cut into 1–2 mm pieces and digested using the gentleMACS dissociation kit (Miltenyi Biotec GmBH, 130-093-235). Cells were then either plated on collagen I–coated (Thermo Fisher Scientific, A1048301) μ-Slides (Ibidi, 80826) or lysed for RNA extraction.

### Immunocytochemistry.

Isolated neonatal mouse cardiomyocytes or mouse HL-1 cells were cultured on μ-Slides and fixed with 4% formaldehyde (Thermo Fisher Scientific, 28908) for 10 minutes at room temperature. Subsequently, cells were permeabilized with 0.1% Triton X-100 in PBS for 15 minutes at room temperature. Next, cells were blocked with 2% BSA (Sigma-Aldrich, A7030) and 1% donkey serum (Sigma-Aldrich, D9663) for 1 hour at room temperature, followed by primary antibody incubation at 4°C overnight in a humidified chamber. Cells were washed 3 times with PBS and incubated with secondary antibodies for 1 hour at room temperature. Phalloidin (Invitrogen, 1:200) was added together with the secondary antibodies to visualize the cytoskeleton. Immunostainings were imaged using a Zeiss LSM780 confocal microscope and analyzed using Volocity software 6.5.1.

The following primary and secondary antibodies were used: rabbit anti–troponin T (Abcam, ab45932; 1:1000), rabbit anti–Aurora B (Abcam, ab2254; 1:500), donkey anti-rabbit–Alexa Fluor 555 (Invitrogen, A-31572; 1:200), and donkey anti-rabbit–Alexa Fluor 555 (Invitrogen, A-31573; 1:200).

### RNAScope.

RNAScope (Advanced Cell Diagnostics) on paraffin-embedded heart sections was performed according to the manufacturer’s instructions. Briefly, deparaffinization and rehydration were done as described above. Sections were incubated with the probe against mouse *Malat1*, which spans nucleotides 712–2338 (transcript ID: NR_002847.2) or with a negative control probe (both designed by Advanced Cell Diagnostics). Probes were hybridized for 2 hours at 40°C in a hybridization oven. Signal amplification was achieved by hybridization with amplification probes serially and using tyramide signal amplification reagents provided within the kit. For immunostaining, sections were blocked with 10% donkey serum, 5% skim milk, and 0.1% Triton X-100 in PBS, followed by primary antibody incubation at 4°C overnight. Cells were washed 3 times with PBS and incubated for 1 hour at room temperature. Slides were imaged using a Zeiss LSM780 confocal laser scanning microscope equipped with 40× and 63× objectives.

The following primary and secondary antibodies were used: rabbit anti–sarcomeric α-actinin antibody (Abcam, ab68167; 1:250) and donkey anti-rabbit–Alexa Fluor 555 (Invitrogen, A-31572; 1:200).

### Maintenance of mouse HL-1 cells.

The murine atrial cardiomyocyte cell line HL-1 (Sigma-Aldrich, SCC065) was cultured in Claycomb medium (Sigma-Aldrich, 51800C) supplemented with 10% fetal bovine serum (FBS) (Invitrogen), 100 μg/mL penicillin/streptomycin (Gibco, 15140122), 0.1 mM norepinephrine (Sigma-Aldrich), and 2 mM GlutaMAX (Gibco, 35050061). Cells were cultured in cell culture dishes coated with 0.02% gelatin (Millipore) and 0.1% fibronectin from human plasma (Sigma-Aldrich, F0895) at 37°C and 5% CO_2_.

### Gene silencing and overexpression in mouse HL-1 cells.

For gene silencing experiments, cells were seeded 24 hours before transfection and were transfected with a final concentration of 50 nM LNA or 50 nM siRNA using RNAimax (Invitrogen, 13778075). Sequences of LNA-gapmers (Qiagen) and siRNAs (Sigma-Aldrich) are listed in Supplemental Table 1.

For hnRNP U overexpression experiments, wild-type and mutant hnRNP U expression plasmids were used. Mutated hnRNP U plasmid lacks the RNA binding RGG-box domain (MRGGNFRGGAPGNRGGYNRRGNMPQR). Both plasmids were designed by OriGene Technologies using the pCMV6-AC-HA mammalian expression vector as backbone (OriGene, PS100004). ORF sequences of both plasmids are listed in Supplemental Table 2. Cells were seeded 24 hours before transfection and then transfected with 2.5 μg of hnRNP U wild-type or mutant plasmid per 300,000 cells/well in 6-well plates using Lipofectamine 3000 reagent (Invitrogen, L3000001). hnRNP U overexpression efficiency was validated by RT-qPCR.

### Total RNA isolation from cell culture and RT-qPCR.

RNA isolation from mouse HL-1 cells was done using RNeasy mini kits (Qiagen, 74104), including an on-column DNase digestion step according to the manufacturer’s instructions. Concentration of total RNA was measured using a Nanodrop 2000 spectrophotometer (Thermo Fisher Scientific). For cDNA synthesis, 1 μg of RNA was reverse transcribed using M-MLV reverse transcriptase (Invitrogen, 28025013) and random hexamers (Qiagen, 79236). RT-qPCR was performed on a StepOnePlus Real-Time PCR system (Thermo Fisher Scientific). Mouse ribosomal protein lateral stalk subunit P0 (*Rplp0*) was used for data normalization and relative expression levels were calculated by the 2^–ΔCt^ method. Primer pairs are listed in Supplemental Table 3.

### RNA sequencing.

RNA was isolated as described above. Total RNA and library integrity were verified on LabChip Gx Touch 24 (PerkinElmer). Total RNA (1 μg) was used as input for SMARTer Stranded Total RNA Sample Prep Kit - HI Mammalian (Clontech, 634873). Sequencing was performed on the NextSeq 500 instrument (Illumina) using v2 chemistry with 1 × 75 bp single-end setup. The resulting raw reads were assessed for quality, adapter content, and duplication rates with FastQC (60). Trimmomatic version 0.39 was employed to trim reads after a quality drop below a mean of Q20 in a window of 10 nucleotides (61). Only reads of at least 15 nucleotides were cleared for subsequent analyses. Trimmed and filtered reads were aligned versus the Ensembl mouse genome version mm10 (Ensembl release 101) using STAR 2.7.7a with the parameters “--outFilterMismatchNoverLmax 0.1 --alignIntronMax 200000” (62). The number of reads aligning to genes was counted with featureCounts 1.6.5 from the Subread package (63). Only reads mapping at least partially inside exons were admitted and aggregated per gene. Reads overlapping multiple genes or aligning to multiple regions were excluded. Differentially expressed genes were identified using DESeq2 version 1.30.0 (64). Only genes with a minimum fold change of ±1.5 (log_2_ ±0.59), a maximum Benjamini-Hochberg–corrected *P* value of 0.05, and a minimum combined mean of 5 reads were deemed to be significantly differentially expressed. The Ensembl annotation was enriched with UniProt data (release 24.03.2017) based on Ensembl gene identifiers (activities at UniProt).

### GO term analysis.

GO analysis was performed using the Enrichr database (https://maayanlab.cloud/Enrichr/) (41, 42, 65).

### Immunoprecipitation and Western blotting.

For immunoprecipitations, mouse HL-1 cells were washed 3 times with ice-cold PBS and extracted with ice-cold RIPA buffer (Sigma-Aldrich, R0278; supplemented with protease and phosphatase inhibitors) for 30 minutes on ice. After centrifugation for 15 minutes at 4°C, the supernatant was collected and incubated with antibody-coupled protein G Dynabeads (Invitrogen, 10003D) (50 μL of beads were coupled with 6 μg antibody) overnight at 4°C. Immunoprecipitates bound to the beads were washed 3 times with wash buffer (150 mM NaCl, 0.05% NP-40, 50 mM Tris HCl pH 8.0, supplemented with protease and phosphatase inhibitors) and the proteins were eluted with Laemmli sample buffer. Samples were resolved by SDS-PAGE and transferred to nitrocellulose membranes at 100 V for 30 minutes in 1× transfer buffer (25 mM glycine, 20% methanol, 0.15% ethanolamine). Afterwards, membranes were blocked with 5% nonfat milk powder for 1 hour at room temperature. Rabbit IgG (Millipore, PP64) was used as IgG control and rabbit anti–hnRNP U antibody (Abcam, ab10297) was used for hnRNP U immunoprecipitation experiments. Antibodies for detecting hnRNP U (Abcam, ab10297; 1:1000) and GAPDH (Cell Signaling Technology, 2118; 1:2000) were diluted in 0.05% Tween 20 and 5% nonfat milk powder and incubated together with membranes overnight at 4°C. Membranes were washed 3 times with TBS-T and incubated with anti-rabbit IgG secondary antibody (Amersham, NA934v; 1:2500) for 1 hour at room temperature. After adding chemiluminescence reagent (Millipore, WBKLS0500), blots were imaged using a ChemiDoc Touch imaging system (Bio-Rad).

### Statistics.

All statistical analyses were done using Prism 8 (GraphPad) software (with the exception of RNA sequencing). Data are expressed as mean ± SEM. The Shapiro-Wilk normality test was used to analyze data distribution. For 2-group comparisons, statistical significance was determined using a 2-tailed Student’s *t* test for normally distributed samples and Mann-Whitney *U* test for the not normally distributed samples. For multiple comparisons, statistical significance was determined using 1-way ANOVA with Tukey’s multiple-comparison test. *P* values of less than 0.05 were considered significant.

### Data availability.

The RNA sequencing data sets generated in this study are available at the NCBI Gene Expression Omnibus database (GEO GSE200733). Source data are provided with this paper.

### Study approval.

All animal procedures were conducted according to the principles of laboratory animal care, institutional guidelines, and approved by local animal ethics Tierschutzbeauftragte from Goethe University Frankfurt. All animal experiments were conducted under permissions FU/1183 and FU/2035 granted by the Regierungspräsidium Darmstadt, Hessen.

## Author contributions

GSA contributed to conceptualization of the study, data curation, formal data analysis, carrying out the investigation, and methodology. NJ contributed to conceptualization, data curation, carrying out the investigation, supervision, and writing the original draft of the manuscript. YM contributed to conceptualization. YF, MS, L Kettenhausen, L Kirchhof, and AF contributed to carrying out the investigation and methodology. SG contributed to carrying out the investigation, methodology, and RNA sequencing. GL contributed to conceptualization, data curation, carrying out the investigation, supervision, writing the original draft of the manuscript, and project administration. SD contributed to conceptualization, supervision, funding acquisition, writing the original draft of the manuscript, and project administration.

## Supplementary Material

Supplemental data

Supplemental video 1

Supplemental video 2

Supplemental video 3

Supplemental video 4

## Version 1 03/08/2023

Electronic publication
