# Supplementary figures and images for: Malat1 deficiency prevents neonatal heart regeneration by inducing cardiomyocyte binucleation
Source: JCI Insight. 2023 Mar 8;8(5):e162124. doi: 10.1172/jci.insight.162124 (PMC10077484; doi:10.1172/jci.insight.162124)

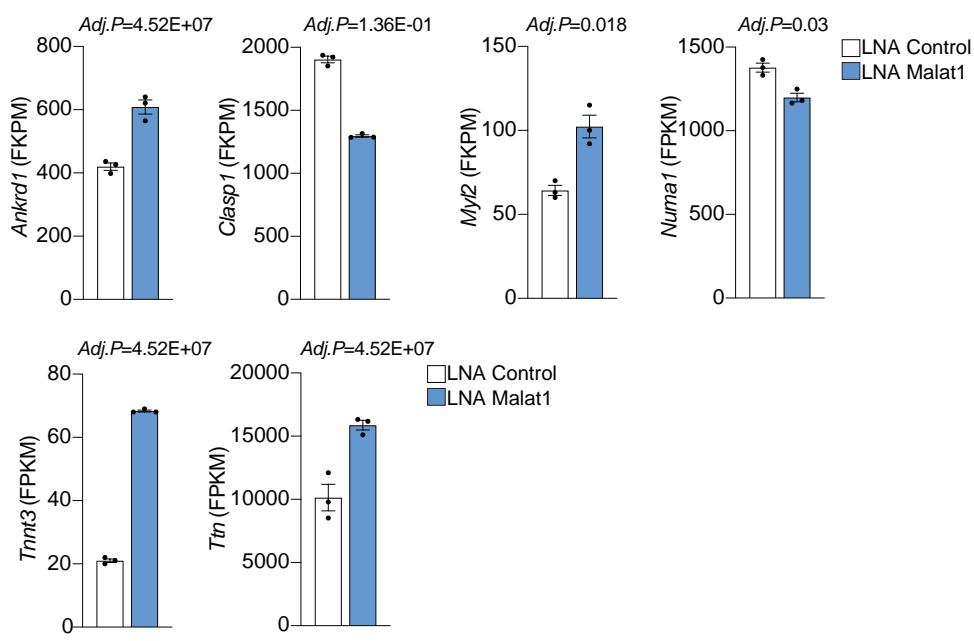

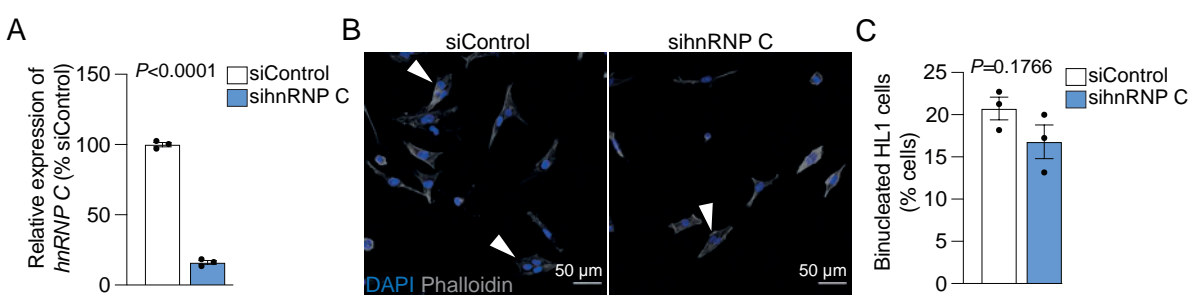

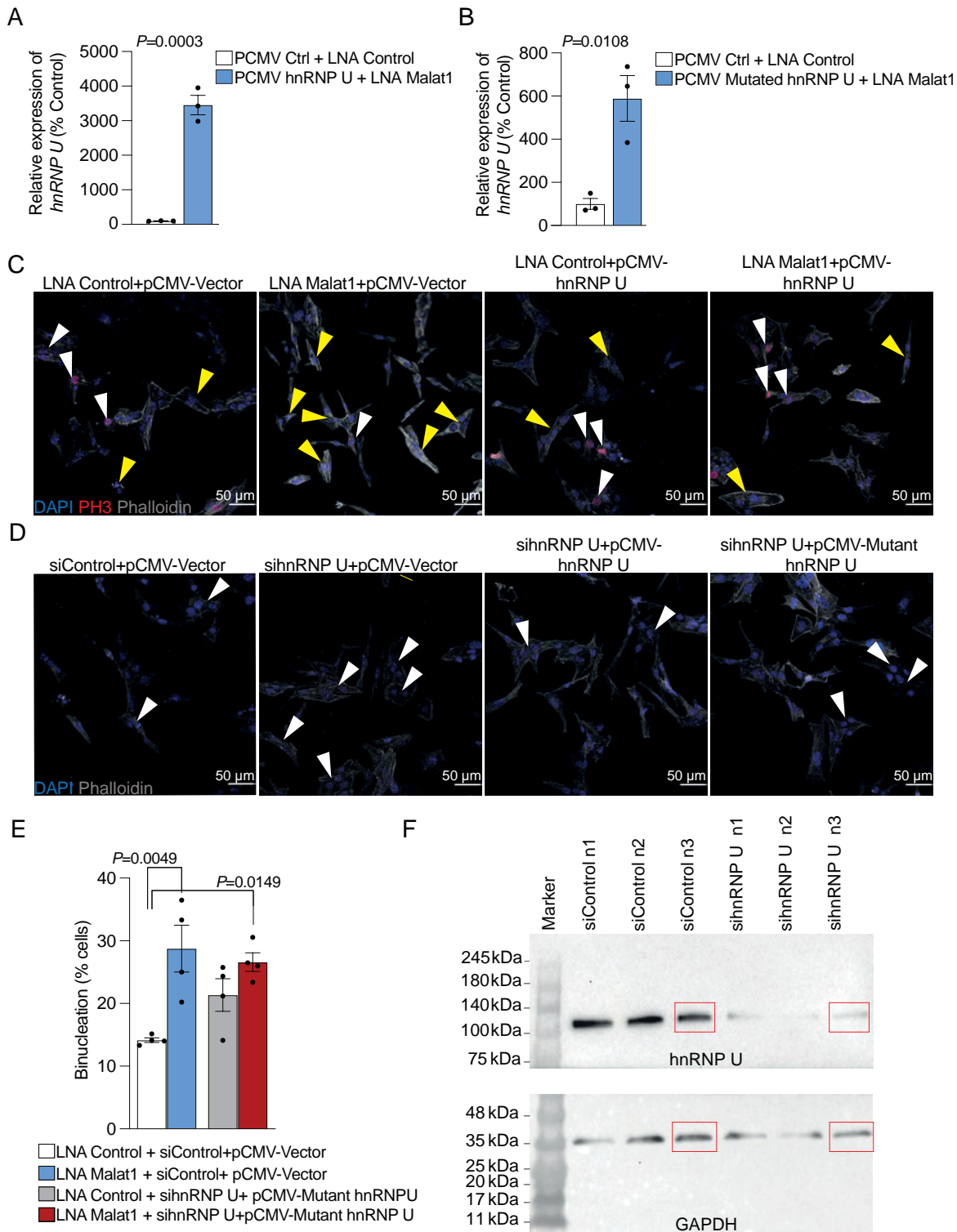

Supplement: Supplemental data [file jciinsight-8-162124-s092.pdf]
